# Young adults, particularly young women, account for an increasingly large share of Dutch mental healthcare expenditure over the period between 2015 and 2021

**DOI:** 10.1017/S2045796024000404

**Published:** 2024-10-11

**Authors:** L. Dijkstra, S. Gülöksüz, A. Batalla, J. van Os

**Affiliations:** 1 Department of Psychiatry, University Medical Centre Utrecht, Utrecht, The Netherlands; 2 Department of Psychiatry, Yale University, New Haven, CT, USA; 3 Department of Psychiatry and Psychology, Maastricht University Medical Centre, Maastricht, The Netherlands

**Keywords:** Epidemiology, Gender Differences, Health Economics, Mental Health, Social and Political Issues

## Abstract

**Aims:**

There is increasing concern over the mental distress of youth in recent years, which may impact mental healthcare utilisation. Here we aim to examine temporal patterns of mental healthcare expenditures in the Netherlands by age and sex in the period between 2015 and 2021.

**Methods:**

Comprehensive data from health insurers in the Netherlands at the 3-number postal code level were used for cluster weighted linear regressions to examine temporal patterns of mental healthcare expenditure by age group (18–34 vs 35–65). The same was done for medical specialist and general practitioner costs. Additionally, we examined interactions with gender, by adding the interaction between age, year and sex to the model.

**Results:**

Mental healthcare costs for younger adults (18–34) were higher than those for older adults (35–65) at all time points (β = 0.22, 95%-CI = 0.19; 0.25). Furthermore there was an increase in the strength of the association between younger age and mental healthcare costs from β = 0.22 (95%-CI = 0.19; 0.25) in 2015 to β = 0.37 (95%-CI = 0.35; 0.40) in 2021 (*p* < 0.0001) and this was most evident in women (*p* < 0.0001). Younger age was associated with lower general practitioner costs at all time points, but this association weakened over time. Younger age was also associated with lower medical specialist costs, which did not weaken over time.

**Conclusions:**

Young adults, particularly young women, account for an increasing share of mental healthcare expenditure in the Netherlands. This suggests that mental distress in young people is increasingly met by a response from the medical system. To mitigate this trend a public mental health approach is needed.

## Introduction

In recent years, there has been a growing concern about worsening youth mental health, with rising rates of depression, anxiety and other mental health issues among younger populations.(Hoffower and Akhtar, 2019; Oladipo, 2023; Patton *et al.*, 2016; The Economist, 2018) Factors such as the pervasive threat of climate change, dwindling biodiversity, individualisation of society, economic inequality and the unprecedented impact of the COVID-19 pandemic have been thought to be significant contributors to this trend. (Christensen *et al.*, 2017; Clayton, 2021; Hossain *et al.*, 2020; Jones *et al.*, 2021; Lund *et al.*, 2018; Mehta *et al.*, 2015; Palinkas and Wong, 2020; Santos *et al.*, 2017) Mental disorders are known to emerge at a young age and cause a higher burden of disease in younger people compared to older people globally. (Fusar-Poli, 2019; GBD 2019 Mental Disorders Collaborators, 2022; Kessler *et al.*, 2005; Solmi *et al.*, 2022) Studies from mostly high-income countries have investigated trends in the prevalence of mental health conditions in younger adults over time. For instance, in the Netherlands, an increase in the prevalence of common mental disorders in people aged 18–34 has been observed between 2007–2009 and 2019–2022. (ten Have *et al.*, 2023) Two studies performed in the United Kingdom observed similar trends, with a records based cohort study showing an increase in the incidence of common mental disorders in the period between 2000 and 2020 in people aged 16–24, (Dykxhoorn *et al.*, 2023) and another records based cohort study showing an increase in anxiety among young people over the period between 2003 and 2018. (Archer *et al.*, 2022) In Belgium, a study among a population based sample of youth aged 15–25 years showed an increase in anxiety and depression between 2008 and 2013. (Van Droogenbroeck *et al.*, 2018) In the United States data from a population based survey showed an increase in moderate depression among those aged 20–39 between 2005 and 2016. (Yu *et al.*, 2020) Many reports have also shown an increase in mental health problems among adolescents under 18 years of age, with evidence suggesting that gender plays a significant role. (Collishaw, 2015; Högberg *et al.*, 2020; Potrebny *et al.*, 2017; Van Droogenbroeck *et al.*, 2018).

The epidemiology of mental health disorders in young people and adolescents is known to differ by gender, with women having a higher burden of disease in this age group. (Van Droogenbroeck *et al.*, 2018; World Health Organization, 2004) A recent report from the Netherlands showed that women aged 16–25 years more often reported mental health problems, loneliness, and suicidal thoughts in 2022 compared to young men.(RIVM *et al.* 2023) It has also been repeatedly reported that female gender is associated with more mental health service utilisation. (Lin *et al.*, 1996; Rhodes *et al.*, 2002; Smith *et al.*, 2013; Vasiliadis *et al.*, 2005; Wang *et al.*, 2005) Men however are known to have more substance use disorders. (Seedat *et al.*, 2009).

Mental health service utilisation is known to be strongly associated with incidence and prevalence of mental disorders and mental suffering, including in the Netherlands.(Boerema *et al.*, 2017; ten Have *et al.*, 2001) Mental health service expenditures thus represent a mix of both incidence and prevalence of mental health problems as a proxy, although not all people with mental health problems will seek help or will be able to find help. Healthcare expenditure and resource utilisation can also be a topic of interest itself. In the Netherlands, data on healthcare expenditure collected by health insurers per postal code area is publicly available with expenditures stratified by area, calendar year, sex and age. As an earlier report on the rise in common mental health conditions in youth in the Netherlands was only available by sampling at two time points, longitudinal records based on health insurance data would be able to offer a more fine-grained image of this rise over time. (ten Have *et al.*, 2023) The use of insurance data is also less prone to sampling bias which could add to the knowledge base on youth and young adults’ mental health in the Netherlands with the study by ten Have et al. having an underrepresentation of younger people. (ten Have *et al.*, 2023) Additionally, analysing patterns of mental healthcare costs would give insight into whether the previously reported rise over time also results in increased service use. This is an area of concern, as the recent increase in prevalence of mental disorders in young people is thought to be the result of socioeconomic and existential factors requiring a public mental health response. (van Os and Guloksuz, 2024) In the absence of an orchestrated public mental health response in the Netherlands, however, we hypothesized that there would be an increase in mental health expenditure in young adults aged 18–34 over recent years and that there would also be a relative increase in mental health expenditure compared to older groups. (van Os *et al.*, 2024) We additionally hypothesised that this effect would be stronger in women. Additionally, we explored the relationship between young age and medical specialist care costs and primary care costs in order to examine the possibility of general trends across all health outcomes, not specific to mental health. We also explored whether patterns in expenditure were different for different age groups within the group of younger adults.

## Methods

### Dataset and measures

Data from Vektis were used, which registers all health insurance costs nationally. (Vektis, 2022) In the Netherlands, adult mental healthcare is paid through the health insurance companies, with the exception of scarcely used private out-of-pocket mental healthcare. All citizens are required by law to be insured; therefore, health insurance data cover almost the entire population, with very few exceptions. People with a low income get a subsidy from the government to pay for health insurance. Mental healthcare costs in the insurance data relate to costs associated with all mental health conditions, such as depression, psychosis, substance use disorders, developmental disorders and personality disorders. Mental healthcare costs include care by a range of professions, such as doctors, nurses, psychologists and social workers. (Vektis, 2023) Protected living facilities and other social care facilities are not paid for by health insurers but by municipal social care programmes and are therefore not included in the data. Health insurers report the costs for clinical stay for a period longer than a year in a mental healthcare facility separately. (Vektis, 2023) Since these stays often occur in specialised, centralised clinics, where patients may also register the clinic’s address as their own, these costs were excluded from the analysis. This exclusion helps prevent potential misrepresentation of the association between costs and postal code area variables. The unit of analysis was the first 3-number postal code area (PC-3) out of the total 6 digit postal code area subdivided by age in years and sex, meaning that each observation is a group of people living in the same postal code area of the same age and of the same sex in the same calendar year. Individual level data, 4-number or 6-number postal code area data are not publicly available for privacy reasons. Similarly, units of analysis smaller than 10 people are removed from the publicly available datasets or are combined with part of the identifying data such as age and postal code removed for privacy reasons. (Vektis, 2023) The group aged 18–65 years was included for analysis. As mental healthcare for children is not paid for by health insurers but through municipal youth mental healthcare programmes, no data were available for people younger than 18 years. People above the age of 65 years were not included to prevent age-specific effects of neuropsychiatric conditions to possibly obscure the results. For the main analysis, age was dichotomized with a cut-off of 35 years. As there is no universal definition on what ages constitute youth or young adulthood, this was done in consistency with a previous report about the rise in common mental health conditions among youth in the Netherlands. (ten Have *et al.*, 2023; United Nations, 2023) In order to conduct exploratory analysis of age differences in trends in mental healthcare expenditure within the group of young adults, an additional variable with age bands 18–19, 20–24, 25–29, 30–24 and 35–65 was created. Sex was the sex that people were registered with at the insurance company, which corresponds to the sex in one’s passport or ID card. Data for the years 2015–2021 was used. Although insurance data are available from the year 2011 onwards, the years 2011–2014 were not used as insurance data for these years are incomplete and unreliable. It should be noted that the dataset only contains costs that were already paid for by the insurer and costs that were not yet invoiced by the service providers are not included. Late invoicing is more common in mental healthcare, meaning the costs in the final year of the dataset might be only 90% complete. (Vektis, 2023) To our knowledge there is no evidence of different billing practices by age. The dataset was merged with public datasets from the Central Bureau for Statistics (CBS) on the level of urbanicity of each postal code. (Centraal Bureau voor de Statistiek, 2015a) Urbanicity is defined by the density of addresses per square kilometre and divided into 5 categories ranging from low to high urbanicity by the CBS. Urbanicity was examined as a confounder in the analysis as it is known to correlate with the prevalence of mental health conditions and with a younger population. (Eurostat, 2022; Penkalla and Kohler, 2014) Although data on various socio-economic indicators is available by postal code area, this was not used as a confounder in this analysis as socio-economic status at the PC-3 area level was not considered meaningful due to the size and heterogeneity within such areas.

The main outcome measure used was the average mental healthcare costs per insured year within a unit of analysis. Additionally, total healthcare costs, general practitioner costs and medical specialist costs per PC-3 aggregated insured person-year were used. General practitioner costs are all costs related to care in the general practitioners practice and include consultations with the doctors, with an assistant and with a ‘praktijkondersteuner’, which is a specialised assistant that sometimes focusses on specific conditions. (Vektis, 2023) Many general practitioners also hire an ‘praktijkondersteuner’ that specifically focusses on mental health and costs related to this are therefore included in the general practitioner costs. Medical specialist costs include all costs made in hospital care, such as consultations with doctors and the care of nurses, as well as other curative healthcare, such as surgeries in specialised centres. (Vektis, 2023) Age, sex, urbanicity and calendar year were the other variables used.

### Analysis

Analyses were conducted using Stata version 18. (StataCorp, 2023) Means, standard deviations, medians and interquartile ranges for the healthcare costs were calculated by age group to describe the data. Data were checked for missingness. Histograms, symmetry plots and quantile plots were made to assess the distribution of healthcare costs. Healthcare costs underwent a zero-skewness-logtransformation, were centred and expressed in standard deviation units for analysis of associations with the predictor variables when not normally distributed. To verify urbanicity was a potential confounder in our sample cross-tabulations of the number of insured people by age and urbanicity were made and mean log-transformed mental healthcare costs weighted by the number of insured people were calculated by urbanicity.

A linear regression of mental healthcare costs and age weighted for the number of insured years per unit of analysis and accounting for urbanicity, sex, year, the interactions between age and year was done. The regression accounted for clustering by PC-3 using cluster-robust standard errors, which allows for the use of analytic weights. Marginal estimates for the age groups were calculated for this model and plotted by year to visualise these results. The same was done with a variable with smaller age bands using 35–65 as the base to explore different patterns in expenditure within the group of young adults. Additionally, the marginal effect of age on mental healthcare costs by year was estimated and plotted. As a new system of imbursement was introduced on 1 January 2022, there were more registration issues in 2021 compared to other years. Thus a sensitivity analysis without the year 2021 for the linear regression of mental healthcare costs and age was done and marginal estimates for the age groups were plotted by year for this regression. Similar linear regressions were applied for general practitioner costs and medical specialist costs and the marginal effect of age on those healthcare costs was also calculated and plotted by year. To explore the effect of sex a linear regression of mental healthcare costs and sex weighted for the number of insured years per unit of analysis and accounting for urbanicity, age, year, the interactions between sex and year was done and the marginal estimates for sex were plotted. To test our hypothesis regarding the effect of sex on the relationship between age and mental healthcare costs, the linear regression for mental healthcare costs was repeated with the threeway-interaction between age, cost and sex and the marginal effects of the variables were calculated and plotted. Wald-tests were used to assess the hypotheses in all the models and reported *p*-values for interactions are for the whole interaction term.

## Results

### Descriptives

There were a total of 515051 units of analysis. The average number of people per unit were 145 and all units of analysis combined represented a total of 73868937 person-years. For the year 2015 the dataset represented 98.87% of the total population aged 18–65.(Centraal Bureau voor de Statistiek, 2015b) The mean total healthcare expenditure per insured year lived in the sample was 2503.59 euros (SD = 6067.08) ranging from −937.23 euros to 481961.80 euros with a median of 1762.45 euros (IQR = 1108.59; 2581.45). The mean mental healthcare expenditure per insured year lived was 240.43 euros (SD = 354.78) ranging from 0 to 20565.97 euros with a median of 149.60 euros (IQR = 55.54; 305.75). Table 1 shows the mean and median costs in euros per insured year by age group. All healthcare costs were non-normally distributed and right-skewed, so all costs were log-transformed, standardized and centred for further analysis. There was no missing data for any of the variables used in this analysis. There were 4 observations that had negative values for the total healthcare cost in the year 2021, these are likely registration artefacts. Urbanicity was found to be associated with both mental healthcare costs and age (A1).

**Table 1.** Healthcare expenditure in euros per year lived by age group

		Age group	
		18−34 years (*N* = 180252)	35−65 years (*N* = 334799)
Costs			
Total healthcare costs	Mean (SD)	2075.38 (8179.58)	2734.14 (4522.65)
	Median (IQR)	1263.45 (820.57; 2084.97)	1987.80 (1365.60; 2826.92)
Mental healthcare costs	Mean (SD)	303.18 (435.99)	206.65 (296.70)
	Median (IQR)	187.75 (79.19; 380.22)	128.70 (47.00; 265.61)
General practitioner costs	Mean (SD)	152.75 (33.96)	182.80 (43.95)
	Median (IQR)	148.71 (128.61; 171.91)	176.96 (150.85; 209.06)
Medical specialist costs	Mean (SD)	677.71 (626.98)	1228.16 (881.58)
	Median (IQR)	504.48 (316.07; 898.33)	1055.32 (666.97; 1568.09)

### Mental healthcare costs and age

**Figure 1.**
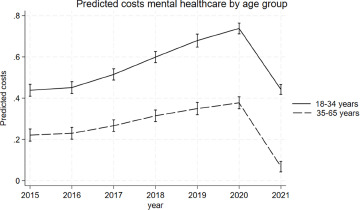
Predicted mental healthcare costs by age group accounting for urbanicity, sex, year, the interaction between age and year. The y-axis shows the log-transformed standardised costs. The X-axis shows the calendar year.

Over the observed period mental healthcare costs increased in both younger and older adults except for a drop between in 2021. This is an artificial drop as a new system of reimbursement was introduced from January first 2022 causing registration issues in the transition year. (Nederlandse Zorgautoriteit, 2021) The marginal prediction for mental healthcare costs was higher for younger adults aged 18–34 compared to older adults aged 35–65 at all time points (Fig. 1), with the regression coefficient for age being 0.22 (95%-CI = 0.19; 0.25). The appendix shows the predicted costs by age group at each time point and the full regression output (A2, A3). Exploratory analysis with smaller age bands showed similar costs between the ages 20–34, but lower costs for 18–19 year olds. The regression coefficient for 18–19 was 0.04 (−0.00; 0.09), while it was 0.20 (0.16; 0.24), 0.25 (0.21; 0.29), 0.27 (0.24; 0.29) for age bands 20–24, 25–29 and 30–34 respectively. (A4, A5, A6).

**Figure 2.**
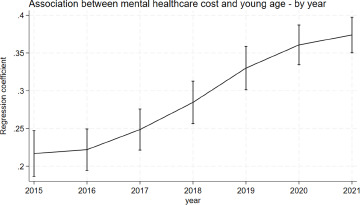
Marginsplot of the association between mental healthcare costs and young age by year. The Y-axis shows the regression coefficient for age. The X-axis shows the calendar year.

The analysis showed evidence for an interaction between young age and year (*p* < 0.0001), with the association between mental healthcare costs and young age increasing over the observed period from β = 0.22 (95%-CI = 0.19; 0.25) in 2015 to β = 0.37 (95%-CI = 0.35; 0.40) in 2021 (Fig. 2). The appendix shows the marginal estimates at each time point (A7). The sensitivity analysis showed similar results for the regression and marginal prediction over the years 2015–2020. (A8, A9, A10).

### Medical specialist and general practitioner costs and age

The regressions and marginsplots for the association between younger age and medical specialist and general practitioner costs by year showed that younger age was associated with lower GP costs at all time points (β = −0.73, 95%-CI = −0.76; -0.71), but that there was evidence for an interaction with age and year (*p* < 0.0001). The strength of the negative association between age and general practitioner costs decreased slightly over the observed period from −0.73 (95%-CI = −0.76; -0.71) in 2015 to −0.63 (95%-CI = −0.65; -0.60), suggesting younger adults accounting for an increasing share of the GP costs over the observed period. Younger age was also associated with lower medical specialist costs (β = −0.91, 95%-CI = −0.92; -0.89) and there was evidence for an interaction with age and year (*p* < 0.0001), with the strength of the negative association increasing slightly over the observed period from β = −0.91 (95%-CI = −0.93; -0.89) in 2015 to β = −0.97 (95%-CI = −0.99; -0.95) in 2021. Appendix A11 to A16 show the regression output, margins plots, and marginal estimates for these analyses.

### Mental healthcare costs, age, and sex

**Figure 3.**
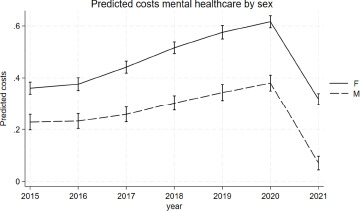
Predicted mental healthcare costs by sex accounting for urbanicity, age, year, the interaction between sex and year. The y-axis shows the log-transformed standardised costs. The X-axis shows the calendar year.

The marginal prediction for mental healthcare costs was higher for women than men at all time points (Fig. 3), with the regression coefficient for sex being β = 0.13 (95%-CI = 0.12; 0.15). The appendix shows the predicted costs by sex at each time point and the full regression output (A17 and A18).

**Figure 4.**
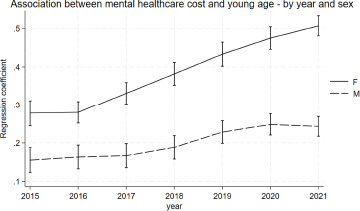
Marginsplot of the association between young age and mental healthcare costs by year and sex. The Y-axis shows the regression coefficient for age. The X-axis shows the calendar year.

There was evidence for an interaction between age, calendar year and sex, with women having a stronger increase in the association between age and mental healthcare cost over the years (*p* < 0.0001 for the three-way-interaction). (Fig. 4) The strength of the association between age and mental healthcare costs increased from 0.28 (95%-CI = 0.25; 0.31) in 2015 to 0.51 (95%-CI = 0.48; 0.53) in 2021 for women, while for men it increased from 0.15 (95%-CI = 0.12; 0.19) in 2015 to 0.24 (95%-CI = 0.22; 0.27) in 2021. Women also had an overall stronger association between young age and mental healthcare costs at all time points (*p* < 0.0001 for the two-way-interaction). The appendix contains an overview of the plotted marginal estimates and of the full regression output (A19 and A20).

## Discussion

The present analysis showed that, over the period of 2015–2021, the association between young age and mental healthcare expenditure increased at the PC-3 level. There was a similar effect for general practitioner costs and a milder opposite effect for medical specialist costs. The increase in the association between age and mental healthcare costs was stronger for women than men.

Our findings are in line with previous records-based and population based studies in high income countries showing worsening youth mental health and show that in the case of the Netherlands this also translates to more mental healthcare consumption. (Archer *et al.*, 2022; Dykxhoorn *et al.*, 2023; ten Have *et al.*, 2023; Van Droogenbroeck *et al.*, 2018) A variety of stressors have been identified as contributing factors. (Clayton, 2021; Hossain *et al.*, 2020; Jones *et al.*, 2021; Lund *et al.*, 2018; Mehta *et al.*, 2015; Palinkas and Wong, 2020; Santos *et al.*, 2017) Although COVID-19 has been one of the factors that were initially thought to have caused this rise, the present analysis suggests that the rise during the pandemic was a continuation of an already existing trend and therefore perhaps not attributable to the pandemic. (Ahmed *et al.*, 2023; Hossain *et al.*, 2020; Panchal *et al.*, 2023; ten Have *et al.*, 2023) However it should be noted that some mental healthcare providers began to offer more online consultation during the pandemic, which could have been more accessible to younger people compared to older people, thus possibly increasing the strength of the association between younger age and mental health expenditure we found in our data. It could be argued that experiencing the current pressures of individualisation and (intergenerational) income inequality in the formative period of adolescence and early adulthood along with the prospects of a bleaker future than previous generations due to climate change lead to the rise in the association between mental healthcare expenditure and young age we observed. (Clayton, 2021; Lund *et al.*, 2018; Mehta *et al.*, 2015; Palinkas and Wong, 2020; Santos *et al.*, 2017; Spitzer *et al.*, 2023) Additionally, in the case of increased mental healthcare consumption it has also been hypothesised that there is a societal trend towards medicalisation of mental suffering. (Aneshensel *et al.*, 2013; Beeker *et al.*, 2021; Foulkes and Andrews, 2023) It could thus be speculated that the aforementioned pressures on young people – apart from increasing the risk of mental illness – also increase the tendency to seek professional help and professional diagnosis as this may have the appealing connotation of fixability. (Lewis, 2022) The trends in mental healthcare costs we observed in the Netherlands might be generalizable to other high-income countries with similar mental health systems, but is not indicative of global trends in expenditure as there is a high unmet need in mental healthcare in low income countries. (Patel *et al.*, 2007; Thornicroft, 2007; Wang *et al.*, 2007).

Our exploratory finding showing mental healthcare costs were lower for 18–19 year olds compared to 20–34 year olds was surprising. However, this may be due to the way the mental health system is organised in the Netherlands, as those under the age of 18 will receive mental healthcare from different organisations than those over the age of 18, meaning that once young people reach the age of 18 they have to be referred to different services which sometimes leads to discontinuation of care. (Gerritsen *et al.*, 2020; van Amelsvoort, 2014) As this was an exploratory analysis, more research needs to be done to interpret these results.

The observed increase in the association between age and general practitioner costs might be due to mental health issues in this age group too. In the Netherlands, access to mental health services requires a referral from a general practitioner. In some instances, a general practitioner might also offer limited treatment for mental health issues or employ a ‘praktijkondersteuner’ to do so. In 2014 the mental healthcare system in the Netherlands was reformed in an attempt to reduce costs so that only people meeting the criteria of a DSM diagnosis could be treated in mental healthcare services and psychological treatment of less complex cases had to be done with a limited number of sessions. (Verhaak *et al.*, 2015) This means that more social and mental health problems were managed within a general practice after 2014, which could be part of the reason why we observed a similar trend in costs in general practice compared to mental healthcare. This explanation is supported by the fact that there was no increase in the association between young age and medical specialist costs, since an increase in youths’ share in those costs might also be expected if younger adults visited their general practitioner more for physical issues and thus got more referrals. However, it could be that if the changes in the mental healthcare system led to different referring practices by age this could have contributed to the trends we observed for mental healthcare costs.

Our finding on the sex differences in mental healthcare expenditures is in line with previous reports of young women reporting more mental health problems than young men and female sex being associated with mental health service utilisation. (Lin *et al.*, 1996; Rhodes *et al.*, 2002; RIVM, GGD, Nivel and ARQ Nationaal Psychotrauma Centrum, 2023; Smith *et al.*, 2013; Van Droogenbroeck *et al.*, 2018; Vasiliadis *et al.*, 2005; Wang *et al.*, 2005; World Health Organization, 2004) Our study only had data on sex as register in a person’s passport and no data on self-reported gender. Assuming sex is often associated with assigned gender roles, sex could be seen as a proxy for gender in our study.

Although our study related to an older age group, our study is also in line with a 2015 review of studies from European countries, New Zealand, and Brazil that showed the long term increase in children and adolescents with emotional problems was stronger for girls than boys in most studies. (Collishaw, 2015) Producing plausible hypotheses on the reasons behind the rise in mental healthcare consumption among young women compared to young men in the Netherlands in recent years presents a complex yet intriguing endeavour. One possibility is that increasing awareness of gender inequalities over recent years has empowered women to seek help more readily. Alternatively, the mental health of young women may have deteriorated due to persisting gender inequalities and gender norms intersecting with newer stressors such as an increasing individualistic and competitive society. (Wiklund *et al.*, 2010) It should be noted that these hypotheses are probably very context specific and might only apply to countries with a similar culture to the Netherlands.

It should however be recognized that gender norms can affect the mental health of men and boys as much as they affect women and girls. Norms on masculinity could make men less likely to seek help for mental health problems such as depression. (Kessler *et al.*, 1981; Moller-Leimkuhler, 2002) Gender may also affect the reaction to trauma and psychosocial suffering, with men being more likely to show externalising behaviours. (Chandy *et al.*, 1996; Wålinder and Rutz, 2001) It has even been proposed that depression is under-detected as it presents differently in men with more externalising symptoms such as substance abuse and decreased impulse control. (Affleck *et al.*, 2018; Bilsker *et al.*, 2018; Oliffe *et al.*, 2019) This is supported by the fact that globally men are more likely to have a substance use disorder.(Seedat *et al.*, 2009) Given that the mental healthcare system often has less capacity to treat addictions and externalising symptoms, this could in part explain the stronger association between age and mental healthcare costs in women. (de Nederlandse ggz, 2022; Nederlands Zorgautoriteit, 2024; Wisselink *et al.*, 2024) Our findings on lower mental health expenditures in young men compared to young women could be indicative of an unmet need for care among young men. The fact that there are twice as many suicides in men in the Netherlands compared to women also suggests that mental health services might insufficiently reach men. (Traag and Hoogenboezem, 2021) The perspectives of men are also underrepresented in mental health research, as a recent review on the self-perceived needs of adolescents with suicidal behaviour suggests. (Looijmans *et al.*, 2023).

### Strengths and limitations

This study had several limitations. First, as it was an ecological study, no definite conclusions can be made about individual-level associations. As the PC-3 area is a fairly large geographical area, these areas can be heterogeneous in nature and can comprise different neighbourhoods, meaning it was not possible to look at other social, economic and geographical determinants of mental health expenditure. Second, this study only examined costs, which is known to be associated with burden of disease for mental health problems but is not necessarily the same. (Boerema *et al.*, 2017; ten Have *et al.*, 2001) It could for instance also be the case that young people were offered more costly treatments than older people in recent years when they presented at mental health services. Examining costs only limits the results generalizability of the results to other countries as distribution of resources may also be the result of the specific way in which the mental health system in the Netherlands is organised. A further limitation in examining costs was that no detailed information on costs per diagnosis or cost per type of mental healthcare was available. Third, our sample excluded some marginalised groups that do not fall under the system of insurance, such as refugees and undocumented people. The fact that data on only sex was available, but not on self-reported gender also means that out study fails to include the perspectives of trans or non-binary people, who are known to experience higher levels of poor mental health. (Dhejne *et al.*, 2016; Tebbe and Budge, 2022) A final issue that affects most research on adolescents, youth, young people and young adults is that there is no universally accepted definition of what ages define youth, making it hard to compare between studies. (United Nations, 2023) For instance, the United Nations define youth as those between 15 and 24 years of age, the Dutch CBS defines young people as all those under the age of 25, and the African Union defines youth as the ages 15–35. (African Union, 2006; Centraal Bureau voor de Statistiek, 2023; United Nations, 2023) Our study used the definition 18–35 years for young adults, which in line with a previous report on the mental health of young adults in the Netherlands. (ten Have *et al.*, 2023) We also chose this age range as we were not able to include those under the age of 18 as they are not included in the insurance data.

Strengths of the present study were the comprehensiveness of the sample, which includes most of the Dutch population. Additionally, having data on the exact age and sex of the people living in a postal code area ensured the people within an observation were as homogenous as possible and allowed for making – cautious – inferences about age and sex. Another strength is that this study is longitudinal with data per year offering a detailed insight into time-trends, which to our knowledge had not been done at a national level before.

### Implications

This study contributes to the evidence base for the growing concern about youth mental health and the fact that this may result in medicalization of distress rather than in a public mental health response. To our knowledge this is the first longitudinal study to look at mental healthcare expenditure associated with this rise in the Netherlands. Notably, our findings highlight that young adults, especially young women, represent a growing portion of these costs. While recognising mental health as a fundamental right (Patel, 2023), this study also provides additional economic incentive for governments and insurers to invest in youth mental health through public mental health measures like risk reduction, health promotion and enhancing resilience, targeting social and existential factors that impact youth mental health. Our findings suggest that mental distress in young adults is increasingly met by a medical response. However as the underlying causes of increased mental distress in young adults are likely social and existential, a social and public health response focussing on underlying causes might be more effective but is currently lacking in the Netherlands. (van Gils *et al.*, 2020) Additionally further research with detailed insurance data at a finer geographical level, such as 6-number postal code areas, could be helpful in revealing neighbourhood level factors that influence mental healthcare consumption and could help guide the planning of local-level preventative interventions.

## Supporting information

Dijkstra et al. supplementary material Dijkstra et al. supplementary material

## Data Availability

Stata datasets and do-files are available upon request to the corresponding author. All original datasets used are publicly available via the Dutch Central Bureau for Statistics (https://opendata.cbs.nl/statline#/CBS/nl/) and Vektis (https://www.vektis.nl/open-data).

## Supplementary material

The supplementary material for this article can be found at https://doi.org/10.1017/S2045796024000404.

## References

[ref1] Affleck W, Carmichael V and Whitley R (2018) Men’s mental health: Social determinants and implications for services. *The Canadian Journal of Psychiatry* 63(9), 581–589. 29673270 10.1177/0706743718762388 PMC6109884

[ref2] African Union (2006) *African Youth Charter*. Addis Ababa: African Union.

[ref3] Ahmed N, Barnett P, Greenburgh A, Pemovska T, Stefanidou T, Lyons N, Ikhtabi S, Talwar S, Francis ER, Harris SM, Shah P, Machin K, Jeffreys S, Mitchell L, Lynch C, Foye U, Schlief M, Appleton R, Saunders KRK, Baldwin H, Allan SM, Sheridan-Rains L, Kharboutly O, Kular A, Goldblatt P, Stewart R, Kirkbride JB, Lloyd-Evans B and Johnson S (2023) Mental health in Europe during the COVID-19 pandemic: A systematic review. *The Lancet Psychiatry* 10(7), 537–556. 37321240 10.1016/S2215-0366(23)00113-X PMC10259832

[ref4] Aneshensel CS, Phelan JC and Bierman A (2013) *Handbook of the Sociology of Mental Health*, 2nd edn. Dordrecht: Springer.

[ref5] Archer C, Turner K, Kessler D, Mars B and Wiles N (2022) Trends in the recording of anxiety in UK primary care: A multi-method approach. *Social Psychiatry & Psychiatric Epidemiology* 57, 375–386. 34196743 10.1007/s00127-021-02131-8 PMC8246441

[ref6] Beeker T, Mills C, Bhugra D, te Meerman S, Thoma S, Heinze M and von Peter S (2021) Psychiatrization of society: A conceptual framework and call for transdisciplinary research. *Frontiers in Psychiatry* 12, 645556. 10.3389/fpsyt.2021.645556 PMC8211773 34149474

[ref7] Bilsker D, Fogarty AS and Wakefield MA (2018) Critical issues in men’s mental health. *The Canadian Journal of Psychiatry* 63(9), 590–596. 29673272 10.1177/0706743718766052 PMC6109879

[ref8] Boerema AM, ten Have M, Kleiboer A, de Graaf R, Nuyen J, Cuijpers P and Beekman ATF (2017) Demographic and need factors of early, delayed and no mental health care use in major depression: A prospective study. *BMC Psychiatry* 17, 367. 10.1186/s12888-017-1531-8 PMC5691833 29145820

[ref9] Centraal Bureau voor de Statistiek (2015a) Kerncijfers Wijken en Buurten [Core Figures Districts and Neighbourhoods].

[ref10] Centraal Bureau voor de Statistiek (2015b) Bevolkingscijfers 2015 [Population Figures 2015].

[ref11] Centraal Bureau voor de Statistiek (2023) Landelijke jeugdmonitor 2023 [National Youth Monitor 2023].

[ref12] Chandy JM, Blum RW and Resnick MD (1996) Gender-specific outcomes for sexually abused adolescents. *Child Abuse and Neglect* 20, 1219–1231. 8985612 10.1016/s0145-2134(96)00117-2

[ref13] Christensen H, Reynolds CF and Cuijpers P (2017) Protecting youth mental health, protecting our future. *World Psychiatry: Official Journal of the World Psychiatric Association (WPA)* 16(3), 327–328. 28941108 10.1002/wps.20437 PMC5608817

[ref14] Clayton S (2021) Climate change and mental health. *Current Environmental Health Reports* 8, 1–6. 33389625 10.1007/s40572-020-00303-3

[ref15] Collishaw S (2015) Annual research review: Secular trends in child and adolescent mental health. *Journal of Child Psychology and Psychiatry and Allied Disciplines* 56, 370–393. 25496340 10.1111/jcpp.12372

[ref16] de Nederlandse ggz (2022) Kerncijfers over de Nederlandse ggz [Key Figures on the Dutch Mental Healthcare system].

[ref17] Dhejne C, Van Vlerken R, Heylens G and Arcelus J (2016) Mental health and gender dysphoria: A review of the literature. *International Review of Psychiatry* 28, 44–57. 26835611 10.3109/09540261.2015.1115753

[ref18] Dykxhoorn J, Osborn D, Walters K, Kirkbride JB, Gnani S and Lazzarino AI (2023) Temporal patterns in the recorded annual incidence of common mental disorders over two decades in the United Kingdom: A primary care cohort study. *Psychological Medicine* 54(4), 663–674. 37605881 10.1017/S0033291723002349

[ref20] Eurostat (2022, October) Urban-rural Europe - demographic developments in cities

[ref21] Foulkes L and Andrews JL (2023) Are mental health awareness efforts contributing to the rise in reported mental health problems? A call to test the prevalence inflation hypothesis. *New Ideas in Psychology* 69, 101010.

[ref22] Fusar-Poli P (2019) Integrated mental health services for the developmental period (0 to 25 Years): A critical review of the evidence. *Frontiers in Psychiatry* 10, 355. 10.3389/fpsyt.2019.00355 PMC6567858 31231250

[ref23] GBD 2019 Mental Disorders Collaborators (2022) Global, regional, and national burden of 12 mental disorders in 204 countries and territories, 1990–2019: A systematic analysis for the Global Burden of Disease Study 2019. *The Lancet Psychiatry* 9, 137–150. 35026139 10.1016/S2215-0366(21)00395-3 PMC8776563

[ref24] Gerritsen SE, Dieleman GC, Beltman MAC, Tangenbergh AAM, Maras A, van Amelsvoort TAMJ and van Staa AL (2020) Transitional psychiatry in the Netherlands: Experiences and views of mental health professionals. *Early Intervention in Psychiatry* 14, 684–690. 31747718 10.1111/eip.12890 PMC7687088

[ref25] Hoffower H and Akhtar A (2019) Lonely, burned out, and depressed: The state of millennials’ mental health in 2019. *Bussiness Insider*.

[ref26] Högberg B, Strandh M and Hagquist C (2020) Gender and secular trends in adolescent mental health over 24 years – The role of school-related stress. *Social Science & Medicine* 250, 112890. 10.1016/j.socscimed.2020.112890 32143086

[ref27] Hossain MM, Tasnim S, Sultana A, Faizah F, Mazumder H, Zou L, McKyer ELJ, Ahmed HU and Ma P (2020) Epidemiology of mental health problems in COVID-19: A review. *F1000Research* 9, 636. 10.12688/f1000research.24457.1 PMC7549174 33093946

[ref28] Jones EAK, Mitra AK and Bhuiyan AR (2021) Impact of covid-19 on mental health in adolescents: A systematic review. *International Journal of Environmental Research & Public Health* 18, 2470. 10.3390/ijerph18052470 PMC7967607 33802278

[ref29] Kessler RC, Berglund P, Demler O, Jin R, Merikangas KR and Walters EE (2005) Lifetime prevalence and age-of-onset distributions of DSM-IV disorders in the National Comorbidity Survey Replication. *Archives of General Psychiatry* 62, 593–602. 15939837 10.1001/archpsyc.62.6.593

[ref30] Kessler RC, Brown RL and Broman CL (1981) Sex differences in psychiatric help-seeking: Evidence from four large-scale surveys. *Journal of Health and Social Behavior* 22, 49. 7240706

[ref31] Lewis R (2022) The Urgent problem with seeking psychiatric diagnoses for every problem. *Psychology Today*.

[ref32] Lin E, Goering P, Offord DR, Campbell D and Boyle MH (1996) The use of mental health services in Ontario: Epidemiologic findings. *The Canadian Journal of Psychiatry* 41, 572–577. 8946080 10.1177/070674379604100905

[ref33] Looijmans M, van Bergen D, Popma A, van Eijk N, Mérelle S, van Veen S, Hawton K and Gilissen R (2023) The self-perceived needs of adolescents with suicidal behaviour: A scoping review. *European Child and Adolescent Psychiatry*. 10.1007/s00787-023-02342-1 PMC11805848 38147110

[ref34] Lund C, Brooke-Sumner C, Baron EC, Breuer MPHE, Jordans M, Herrman AH, Lund C, Brooke-Sumner C, Baingana F, Claire Baron E, Breuer E, Chandra P, Haushofer J, Herrman H, Jordans M, Kieling C, Elena Medina-Mora M, Morgan E, Omigbodun O, Tol W, Patel V and Saxena S (2018) Social determinants of mental disorders and the Sustainable Development Goals: A systematic review of reviews. *The Lancet Psychiatry* 5(4), 357–369. 29580610 10.1016/S2215-0366(18)30060-9

[ref35] Mehta K, Kramer H, Durazo-Arvizu R, Cao G, Tong L and Rao M (2015) Depression in the US population during the time periods surrounding the great recession. *Journal of Clinical Psychiatry* 76, e499–e504. 25919842 10.4088/JCP.14m09637

[ref36] Moller-Leimkuhler AM (2002) Barriers to help-seeking by men: A review of sociocultural and clinical literature with particular reference to depression. *Journal of Affective Disorders* 71, 1–9. 12167495 10.1016/s0165-0327(01)00379-2

[ref37] Nederlandse Zorgautoriteit (2021) Informatiekaart Zorgprestatiemodel per 2022 [Informationcard ‘Zorgprestatiemodel’ from 2022]. https://puc.overheid.nl/nza/doc/PUC_646523_22/#:∼:text=Per%202022%20gaan%20we%20in,en%20controleerbaar%20voor%20de%20pati%C3%ABnt (accessed 31 January 2024).

[ref38] Nederlands Zorgautoriteit (2024) Kerncijfers ggz [Key Figures Mental Healthcare].

[ref39] Oladipo G (2023) Mental health of young adults severely impacted by pandemic – Study. *The Guardian*

[ref40] Oliffe JL, Rossnagel E, Seidler ZE, Kealy D, Ogrodniczuk JS and Rice SM (2019) Men’s depression and suicide. *Current Psychiatry Reports* 21, 103. 10.1007/s11920-019-1088-y 31522267

[ref41] Palinkas LA and Wong M (2020) Global climate change and mental health. *Current Opinion in Psychology* 32, 12–16. 31349129 10.1016/j.copsyc.2019.06.023

[ref42] Panchal U, Salazar de Pablo G, Franco M, Moreno C, Parellada M, Arango C and Fusar-Poli P (2023) The impact of COVID-19 lockdown on child and adolescent mental health: Systematic review. Springer Science and Business Media Deutschland GmbH. 10.1007/s00787-021-01856-w PMC8371430 34406494

[ref43] Patel V (2023) The right to mental health. *The Lancet* 402, 1412–1413. 10.1016/S0140-6736(23)02241-9 37813116

[ref44] Patel V, Flisher AJ, Hetrick S and Mcgorry P (2007) Series Mental health of young people: A global public-health challenge. *The Lancet* 369, 1302–1313. 10.1016/S0140-6736(07)60368-7 17434406

[ref45] Patton GC, Sawyer SM, Santelli JS, Ross DA, Afifi R, Allen NB, Arora M, Azzopardi P, Baldwin W, Bonell C, Kakuma R, Kennedy E, Mahon J, McGovern T, Mokdad AH, Patel V, Petroni S, Reavley N, Taiwo K, Waldfogel J, Wickremarathne D, Barroso C, Bhutta Z, Fatusi AO, Mattoo A, Diers J, Fang J, Ferguson J, Ssewamala F and Viner RM (2016) Our future: A Lancet commission on adolescent health and wellbeing. *Lancet* 387(10036), 2423–2478. 27174304 10.1016/S0140-6736(16)00579-1 PMC5832967

[ref46] Penkalla AM and Kohler S (2014) Urbanicity and mental health in Europe: A systematic review. *European Journal of Mental Health* 9, 163–177.

[ref47] Potrebny T, Wiium N and Lundegård MMI (2017) Temporal trends in adolescents’ self-reported psychosomatic health complaints from 1980-2016: A systematic review and meta-analysis. *PLoS ONE* 12, e0188374. 10.1371/journal.pone.0188374 PMC5705135 29182644

[ref48] Rhodes AE, Goering PN, To T and Williams JI (2002) Gender and outpatient mental health service use. *Social Science & Medicine* 54, 1–10. 11820673 10.1016/s0277-9536(01)00002-8

[ref49] RIVM, GGD, Nivel and ARQ Nationaal Psychotrauma Centrum (2023) Corona Gezondheidsmonitor Jongvolwassenen 2022 [Corona Health Monitor Young Adults 2022].

[ref50] Santos HC, Varnum MEW and Grossmann I (2017) Global increases in individualism. *Psychological Science* 28, 1228–1239. 28703638 10.1177/0956797617700622

[ref51] Seedat S, Scott KM, Angermeyer MC, Berglund P, Bromet EJ, Brugha TS, Demyttenaere K, de Girolamo G, Haro JM, Jin R, Karam EG, Kovess-Masfety V, Levinson D, Medina Mora ME, Ono Y, Ormel J, Pennell B-E, Posada-Villa J, Sampson NA, Williams D and Kessler RC (2009) Cross-national associations between gender and mental disorders in the World Health Organization World Mental Health Surveys. *Archives of General Psychiatry* 66, 785–795. 19581570 10.1001/archgenpsychiatry.2009.36 PMC2810067

[ref52] Smith KLW, Matheson FI, Moineddin R, Dunn JR, Lu H, Cairney J and Glazier RH (2013) Gender differences in mental health service utilization among respondents reporting depression in a national health survey. *Health* 05, 1561–1571.

[ref53] Solmi M, Radua J, Olivola M, Croce E, Soardo L, Salazar de Pablo G, Il Shin J, Kirkbride JB, Jones P, Kim JH, Kim JY, Carvalho AF, Seeman MV, Correll CU and Fusar-Poli P (2022) Age at onset of mental disorders worldwide: Large-scale meta-analysis of 192 epidemiological studies. *Molecular Psychiatry* 27, 281–295. 34079068 10.1038/s41380-021-01161-7 PMC8960395

[ref54] Spitzer S, Hammer B and Reiter C (2023) Living conditions and quality of life Intergenerational income dynamics in Europe: Past trends and current challenges Intergenerational differences over time.

[ref55] StataCorp (2023) Stata Statistical Software: Release 18. College Station, TX: StataCorp LLC.

[ref56] Tebbe EA and Budge SL (2022) Factors that drive mental health disparities and promote well-being in transgender and nonbinary people. *Nature Reviews Psychology* 1(12), 694–707 10.1038/s44159-022-00109-0 PMC9513020 36187743

[ref57] ten Have M, Tuithof M, van Dorsselaer S, Schouten F, Luik AI and de Graaf R (2023) Prevalence and trends of common mental disorders from 2007-2009 to 2019-2022: Results from the Netherlands Mental Health Survey and Incidence Studies (NEMESIS), including comparison of prevalence rates before vs. during the COVID-19 pandemic. *World Psychiatry: Official Journal of the World Psychiatric Association (WPA)* 22, 275–285. 37159351 10.1002/wps.21087 PMC10168151

[ref58] ten Have M, Vollebergh W, Bijl RV and de Graaf R (2001) Predictors of incident care service utilisation for mental health problems in the Dutch general population. *Social Psychiatry & Psychiatric Epidemiology* 36, 141–149. 11465786 10.1007/s001270050303

[ref19] The Economist (2018) Generation Z is stressed, depressed and exam-obsessed. https://www.economist.com/graphic-detail/2019/02/27/generation-z-is-stressed-depressed-and-exam-obsessed?utm_medium=cpc.adword.pd&utm_source=google&ppccampaignID=18151738051&ppcadID=&utm_campaign=a.22brand_pmax&utm_content=conversion.direct-response.anonymous&gad_source=1&gclid=CjwKCAiAvoqsBhB9EiwA9XTWGQirkJLzWsRvaN6MVvwijDATsE7oPaEs4ctcoOOqvTw7haBxprv_bRoC78kQAvD_BwE&gclsrc=aw.ds (accessed 20 December 2023).

[ref59] Thornicroft G (2007) Most people with mental illness are not treated. *The Lancet* 370(9594), 807–808. 10.1016/S0140-6736(07)61392-0 17826153

[ref60] Traag T and Hoogenboezem J (2021) Zelfdoding in Nederland: Een overzicht vanaf 1950 [Suicide in the Netherlands: An overview from 1950].

[ref61] United Nations (2023) Global issues: Youth. https://www.un.org/en/global-issues/youth (accessed 31 January 2024).

[ref62] van Amelsvoort TAMJ (2014) De kloof overbruggen [Bridging the Gap]. *Tijdschrift voor Psychiatrie* 56, 638–639. 25327343

[ref63] Van Droogenbroeck F, Spruyt B and Keppens G (2018) Gender differences in mental health problems among adolescents and the role of social support: Results from the Belgian health interview surveys 2008 and 2013. *BMC Psychiatry* 18, 6. 10.1186/s12888-018-1591-4 PMC5763832 29320999

[ref64] van Gils PF, Suijkerbuijk AWM, Polder JJ, de Wit GA and Koopmanschap M (2020) Nederlandse preventie-uitgaven onder de loep [Dutch prevention expenditure examined]. *TSG - Tijdschrift voor Gezondheidswetenschappen* 98, 92–96.

[ref65] van Os J and Guloksuz S (2024) Population Salutogenesis-The Future of Psychiatry? American Medical Association 10.1001/jamapsychiatry.2023.4582 38117509

[ref66] van Os J, Scheepers F, Milo M, Ockeloen G, Guloksuz S and Delespaul P (2024) “It has to be better, otherwise we will get stuck.” A Review of Novel Directions for Mental Health Reform and Introducing Pilot Work in the Netherlands. *Clinical Practice and Epidemiology in Mental Health* 19, 1–17. 10.2174/0117450179271206231114064736 PMC11046893 38680529

[ref67] Vasiliadis H-M, Lesage A, Adair C and Boyer R (2005) Service use for mental health reasons: Cross-provincial differences in rates, determinants, and equity of access. *The Canadian Journal of Psychiatry* 50, 614–619. 16276852 10.1177/070674370505001007

[ref68] Vektis (2022) Open Data Kosten Zorgverzekeringswet [Open Data Costs Healthcare Insurance Act]. https://www.vektis.nl/open-data (accessed 1 October 2023).

[ref69] Vektis (2023) Bijsluiter bij de Vektis Open Databestanden Zorgverzekeringswet 2011–2021.

[ref70] Verhaak PFM, Magnée T, Hooiveld M, ten Veen P and de Bakker D (2015) Gevolgen invoering Basis GGZ voor de psychische en sociale hulpvraag in de huisartspraktijk. [Consequences of the introduction of basic mental healthcare on psychological and social helpseeking in general practice]. Utrecht.

[ref71] Wålinder J and Rutz W (2001) Male depression and suicide. *International Clinical Psychopharmacology* 16, S21–24. 10.1097/00004850-200103002-00004 11349757

[ref72] Wang PS, Aguilar-Gaxiola S, Alonso J, Angermeyer MC, Borges G, Bromet EJ, Bruffaerts R, de Girolamo G, de Graaf R, Gureje O, Haro JM, Karam EG, Kessler RC, Kovess V, Lane MC, Lee S, Levinson D, Ono Y, Petukhova M, Posada-Villa J, Seedat S and Wells JE (2007) Use of mental health services for anxiety, mood, and substance disorders in 17 countries in the WHO world mental health surveys. *The Lancet* 370, 841–850. 10.1016/S0140-6736(07)61414-7 PMC2847360 17826169

[ref73] Wang PS, Lane M, Olfson M, Pincus HA, Wells KB and Kessler RC (2005) Twelve-month use of mental health services in the United States results from the National Comorbidity Survey Replication. *Arch Ge Psychiatry* 62, 629–640. 10.1001/archpsyc.62.6.629 15939840

[ref74] Wiklund M, Bengs C, Malmgren-Olsson EB and Öhman A (2010) Young women facing multiple and intersecting stressors of modernity, gender orders and youth. *Social Science & Medicine* 71, 1567–1575. 20846769 10.1016/j.socscimed.2010.08.004

[ref75] Wisselink D, Kuijpers W, Kerssies J and van der Slink J (2024) Kerncijfers Verslavingszorg 2017-2022 [Key Figures Addiction Healthcare 2017-2022].

[ref76] World Health Organization (2004) *Gender in Mental Health Research*. Geneva: World Health Organization.

[ref77] Yu B, Zhang X, Wang C, Sun M, Jin L and Liu X (2020) Trends in depression among Adults in the United States, NHANES 2005–2016. *Journal of Affective Disorders* 263, 609–620. 31744739 10.1016/j.jad.2019.11.036

