# Supplementary material for: Young adults, particularly young women, account for an increasingly large share of Dutch mental healthcare expenditure over the period between 2015 and 2021
Source: Epidemiol Psychiatr Sci. 2024 Oct 11;33:e48. doi: 10.1017/S2045796024000404 (PMC11561524; doi:10.1017/S2045796024000404)
Supplement: Dijkstra et al. supplementary material [file S2045796024000404sup001.docx]

Table of contents

[A1 Associations urbanicity with mental healthcare costs and age 2](#_Toc167467514)

[A2 Regression output for mental healthcare costs and age 3](#_Toc167467515)

[A3 Marginal estimates for mental healthcare costs by age group 4](#_Toc167467516)

[A4 Regression output for mental healthcare costs and age with smaller age bands 5](#_Toc167467517)

[A5 Marginsplot for predicted mental healthcare costs and age with smaller age bands 7](#_Toc167467518)

[A6 Marginal estimates for mental healthcare costs by age group with smaller age bands 8](#_Toc167467519)

[A7 Marginal estimates for the association between mental healthcare costs and young age 9](#_Toc167467520)

[A8 Regression output for mental healthcare costs and age – excluding 2021 10](#_Toc167467521)

[A9 Marginsplot for predicted mental healthcare costs and age – excluding 2021 11](#_Toc167467522)

[A10 Marginal estimates for mental healthcare costs by age group - excluding 2021 12](#_Toc167467523)

[A11 Regression output for medical specialist costs and age 13](#_Toc167467524)

[A12 Marginsplot for medical specialist costs and age 14](#_Toc167467525)

[A13 Marginal estimates for the association between medical specialist costs and young age 15](#_Toc167467526)

[A14 Regression output for general practitioner costs and age 16](#_Toc167467527)

[A15 Marginsplot for general practitioner costs and age 17](#_Toc167467528)

[A16 Marginal estimates for the association between general practitioner costs and young age 18](#_Toc167467529)

[A17 Regression output for mental healthcare costs and sex 19](#_Toc167467530)

[A18 Marginal estimates for mental healthcare costs by sex 20](#_Toc167467531)

[A19 Regression output for mental healthcare costs and the interaction between age and sex 21](#_Toc167467532)

[A20 Marginal estimates for the association between mental healthcare costs and young age by gender 22](#_Toc167467533)

# A1 Associations urbanicity with mental healthcare costs and age

|  | Age in years | | |  |
| --- | --- | --- | --- | --- |
|  | 18-34 |  |  | 35-65 |
| Urbanicity |  |  |  |  |
| 1 | 28,95% |  |  | 71,05% |
| 2 | 29,71% |  |  | 70,29% |
| 3 | 30,33% |  |  | 69,67% |
| 4 | 33,01% |  |  | 66,99% |
| 5 | 40,06% |  |  | 59,94% |

Percentage of insured people for each age category by level of urbanicity

|  | Log-transformed mental healthcare costs | |
| --- | --- | --- |
|  | Mean | Standard deviation |
| Urbanicity |  |  |
| 1 | -0.19 | 1.00 |
| 2 | 0.01 | 0.90 |
| 3 | 0.20 | 0.81 |
| 4 | 0.47 | 0.69 |
| 5 | 0.71 | 0.53 |

Tabel 1 Means and standard deviations for log-transformed and standardized mental healthcare costs by urbanicity level weighted for the number of insured people

# A2 Regression output for mental healthcare costs and age

|  | Coefficient | Robust std. err. | t | P>\|t\| | [95% conf. interval] |  |
| --- | --- | --- | --- | --- | --- | --- |
| Urbanicity |  |  |  |  |  |  |
| 2 | 0,205451 | 0,031242 | 6,58 | 0 | 0,144126 | 0,266777 |
| 3 | 0,393319 | 0,038733 | 10,15 | 0 | 0,317288 | 0,46935 |
| 4 | 0,655172 | 0,035135 | 18,65 | 0 | 0,586203 | 0,724141 |
| 5 | 0,870916 | 0,036645 | 23,77 | 0 | 0,798983 | 0,94285 |
| Sex | 0,19908 | 0,007497 | 26,55 | 0 | 0,184363 | 0,213796 |
| Age |  |  |  |  |  |  |
| 18-34 years | 0,21689 | 0,015439 | 14,05 | 0 | 0,186583 | 0,247196 |
| Year |  |  |  |  |  |  |
| 2016 | 0,008365 | 0,004746 | 1,76 | 0,078 | -0,00095 | 0,017681 |
| 2017 | 0,045257 | 0,004826 | 9,38 | 0 | 0,035785 | 0,05473 |
| 2018 | 0,093324 | 0,005178 | 18,02 | 0 | 0,083159 | 0,103488 |
| 2019 | 0,128088 | 0,006422 | 19,95 | 0 | 0,115483 | 0,140693 |
| 2020 | 0,156035 | 0,006501 | 24 | 0 | 0,143274 | 0,168797 |
| 2021 | -0,15268 | 0,006976 | -21,89 | 0 | -0,16638 | -0,13899 |
| Age#year |  |  |  |  |  |  |
| 18-34 years#2016 | 0,005035 | 0,007466 | 0,67 | 0,5 | -0,00962 | 0,01969 |
| 18-34 years#2017 | 0,031679 | 0,008063 | 3,93 | 0 | 0,015852 | 0,047506 |
| 18-34 years#2018 | 0,067662 | 0,008107 | 8,35 | 0 | 0,051748 | 0,083575 |
| 18-34 years#2019 | 0,113085 | 0,010329 | 10,95 | 0 | 0,09281 | 0,133361 |
| 18-34 years#2020 | 0,143851 | 0,008416 | 17,09 | 0 | 0,127331 | 0,160371 |
| 18-34 years#2021 | 0,156949 | 0,009816 | 15,99 | 0 | 0,137681 | 0,176217 |
| constant | -0,61735 | 0,030262 | -20,4 | 0 | -0,67675 | -0,55795 |

Regression output the linear regression of mental healthcare costs and age weighted for the number of insured years per unit of analysis and accounting for urbanicity, sex, year, the interactions between age and year, and for clustering by 3-number postal code.

# A3 Marginal estimates for mental healthcare costs by age group

|  | Age 18-35 | Age 35-65 |
| --- | --- | --- |
|  | *Marginal estimate (95%-CI)* | |
| 2015 | 0.44 (0.41;0.47) | 0.22 (0.19;0.25) |
| 2016 | 0.45 (0.42;0.48) | 0.23 (0.20;0.26) |
| 2017 | 0.51 (0.49;0.54) | 0.27 (0.24;0.29) |
| 2018 | 0.60 (0.57;0.63) | 0.31 (0.29;0.34) |
| 2019 | 0.68 (0.65;0.71) | 0.35 (0.32;0.38) |
| 2020 | 0.74 (0.71;0.76) | 0.38 (0.35;0.41) |
| 2021 | 0.44 (0.42;0.47) | 0.07 (0.04;0.09) |

Marginal estimates for mental healthcare costs by agegroup per year. As plotted in figure 1.

# A4 Regression output for mental healthcare costs and age with smaller age bands

|  | Coefficient | Robust std. err. | t | P>\|t\| | [95% conf. interval] |  |
| --- | --- | --- | --- | --- | --- | --- |
| Urbanicity |  |  |  |  |  |  |
| 2 | 0,205179 | 0,031226 | 6,57 | 0 | 0,143885 | 0,266474 |
| 3 | 0,39289 | 0,038692 | 10,15 | 0 | 0,316941 | 0,46884 |
| 4 | 0,653812 | 0,035069 | 18,64 | 0 | 0,584974 | 0,72265 |
| 5 | 0,866873 | 0,036514 | 23,74 | 0 | 0,795199 | 0,938548 |
| Sex | 0,198849 | 0,007504 | 26,5 | 0 | 0,18412 | 0,213578 |
| Age |  |  |  |  |  |  |
| 30-34 years | 0,268462 | 0,012883 | 20,84 | 0 | 0,243173 | 0,293752 |
| 25-29 years | 0,252034 | 0,019383 | 13 | 0 | 0,213987 | 0,290081 |
| 20-24 years | 0,19872 | 0,020101 | 9,89 | 0 | 0,159262 | 0,238178 |
| 18-19 years | 0,04378 | 0,024162 | 1,81 | 0,07 | -0,00365 | 0,09121 |
| Year |  |  |  |  |  |  |
| 2016 | 0,008411 | 0,004741 | 1,77 | 0,076 | -0,0009 | 0,017718 |
| 2017 | 0,045308 | 0,00482 | 9,4 | 0 | 0,035847 | 0,054769 |
| 2018 | 0,093387 | 0,005172 | 18,06 | 0 | 0,083234 | 0,10354 |
| 2019 | 0,128112 | 0,006407 | 19,99 | 0 | 0,115535 | 0,14069 |
| 2020 | 0,156149 | 0,006491 | 24,06 | 0 | 0,143408 | 0,16889 |
| 2021 | -0,15253 | 0,006964 | -21,9 | 0 | -0,1662 | -0,13886 |
| Age#year |  |  |  |  |  |  |
| 30-34 years#2016 | -0,01288 | 0,010589 | -1,22 | 0,224 | -0,03367 | 0,007903 |
| 30-34 years#2017 | 0,006507 | 0,010787 | 0,6 | 0,547 | -0,01467 | 0,027682 |
| 30-34 years#2018 | 0,038444 | 0,011551 | 3,33 | 0,001 | 0,015771 | 0,061117 |
| 30-34 years#2019 | 0,063681 | 0,01133 | 5,62 | 0 | 0,04144 | 0,085922 |
| 30-34 years#2020 | 0,095147 | 0,011381 | 8,36 | 0 | 0,072807 | 0,117487 |
| 30-34 years#2021 | 0,092209 | 0,011711 | 7,87 | 0 | 0,069222 | 0,115197 |
| 25-29 years#2016 | 0,008685 | 0,010521 | 0,83 | 0,409 | -0,01197 | 0,029336 |
| 25-29 years#2017 | 0,046986 | 0,012116 | 3,88 | 0 | 0,023202 | 0,07077 |
| 25-29 years#2018 | 0,073946 | 0,012057 | 6,13 | 0 | 0,050279 | 0,097614 |
| 25-29 years#2019 | 0,113349 | 0,013401 | 8,46 | 0 | 0,087043 | 0,139655 |
| 25-29 years#2020 | 0,159756 | 0,012174 | 13,12 | 0 | 0,135859 | 0,183653 |
| 25-29 years#2021 | 0,159133 | 0,012965 | 12,27 | 0 | 0,133684 | 0,184583 |
| 20-24 years#2016 | 0,013806 | 0,012035 | 1,15 | 0,252 | -0,00982 | 0,03743 |
| 20-24 years#2017 | 0,035654 | 0,012678 | 2,81 | 0,005 | 0,010767 | 0,06054 |
| 20-24 years#2018 | 0,08838 | 0,012485 | 7,08 | 0 | 0,063873 | 0,112886 |
| 20-24 years#2019 | 0,14877 | 0,016244 | 9,16 | 0 | 0,116884 | 0,180657 |
| 20-24 years#2020 | 0,164338 | 0,013109 | 12,54 | 0 | 0,138605 | 0,190071 |
| 20-24 years#2021 | 0,223512 | 0,014377 | 15,55 | 0 | 0,195291 | 0,251734 |
| 18-19 years#2016 | 0,019263 | 0,021089 | 0,91 | 0,361 | -0,02213 | 0,060659 |
| 18-19 years#2017 | 0,046472 | 0,021206 | 2,19 | 0,029 | 0,004845 | 0,088099 |
| 18-19 years#2018 | 0,074778 | 0,020961 | 3,57 | 0 | 0,033632 | 0,115923 |
| 18-19 years#2019 | 0,148351 | 0,02292 | 6,47 | 0 | 0,10336 | 0,193343 |
| 18-19 years#2020 | 0,172261 | 0,023121 | 7,45 | 0 | 0,126875 | 0,217646 |
| 18-19 years#2021 | 0,146349 | 0,022941 | 6,38 | 0 | 0,101316 | 0,191382 |
| constant | -0,61557 | 0,030305 | -20,31 | 0 | -0,67506 | -0,55608 |

Regression output the linear regression of mental healthcare costs and age weighted for the number of insured years per unit of analysis and accounting for urbanicity, sex, year, the interactions between age and year, and for clustering by 3-number post. Age is divided in bands 18-19, 20-24, 25-29, 30-34, 35-65 and 35-65 was used as the base.

# A5 Marginsplot for predicted mental healthcare costs and age with smaller age bands


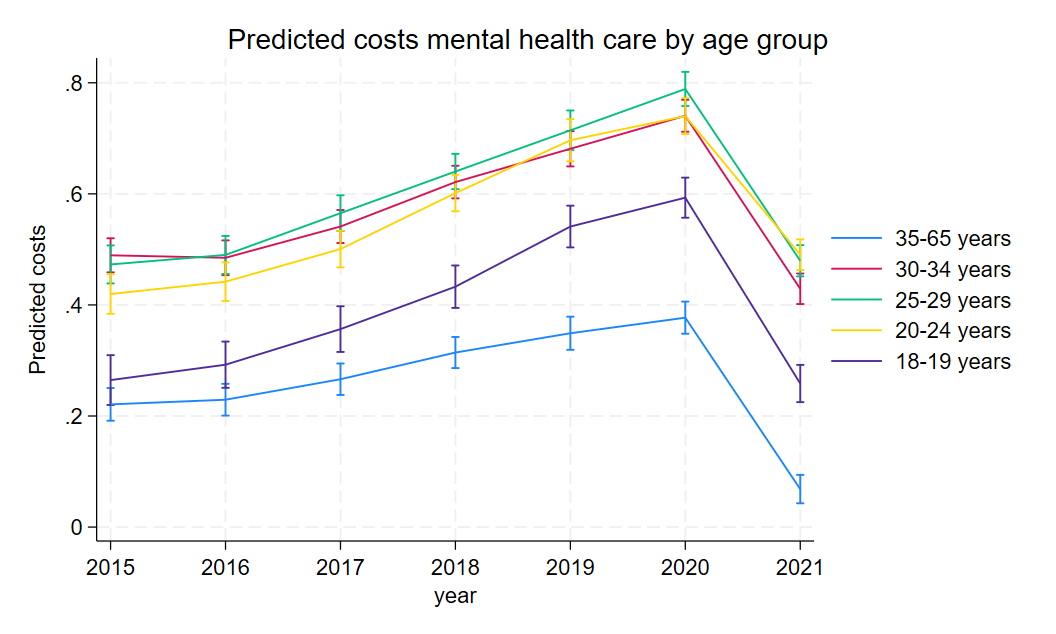


Predicted mental healthcare costs by age group accounting for urbanicity, sex, year, the interaction between age and year. The y-axis shows the log-transformed standardised costs. The X-axis shows the calendar year.

# A6 Marginal estimates for mental healthcare costs by age group with smaller age bands

|  | Age 18-19 | Age 20-24 | Age 25-29 | Age 30-34 | Age 35-65 |
| --- | --- | --- | --- | --- | --- |
|  | *Marginal estimate (95%-CI)* | | | | |
| 2015 | 0.26 (0.22;0.31) | 0.42 (0.38;0.46) | 0.47 (0.44;0.51) | 0.49 (0.46;0.52) | 0.22 (0.19;0.25) |
| 2016 | 0.29 (0.25;0.33) | 0.44 (0.41;0.48) | 0.49 (0.46;0.52) | 0.48 (0.45;0.52) | 0.23 (0.20;0.26) |
| 2017 | 0.36 (0.32;0.40) | 0.50 (0.47;0.53) | 0.57 (0.53;0.60) | 0.54 (0.51;0.57) | 0.27 (0.24;0.29) |
| 2018 | 0.43 (0.39;047) | 0.60(0.57;0.63) | 0.64 (0.61;0.67) | 0.62 (0.59;0.65) | 0.31 (0.29;0.34) |
| 2019 | 0.54 (0.50;0.58) | 0.70 (0.66;0.73) | 0.71 (0.68;0.75) | 0.68 (0.65;0.71) | 0.35 (0.31;0.38) |
| 2020 | 0.59 (0.56;0.63) | 0.74 (0.71;0.77) | 0.79 (0.76;0.82) | 0.74 (0.71;0.76) | 0.38 (0.35;0.41) |
| 2021 | 0.26 (0.23;0.29) | 0.49 (0.46;0.52) | 0.48 (0.45;0.51) | 0.43 (0.40;0.46) | 0.07 (0.02;0.09) |

Marginal estimates for mental healthcare costs by age group per year. As plotted in A5.

# A7 Marginal estimates for the association between mental healthcare costs and young age

|  | *Marginal estimate (95%-CI)* |
| --- | --- |
| 2015 | 0.22 (0.19;0.25) |
| 2016 | 0.22 (0.19;0.25) |
| 2017 | 0.25 (0.22;0.28) |
| 2018 | 0.28 (0.26;0.31) |
| 2019 | 0.33 (0.30;0.36) |
| 2020 | 0.36 (0.33;0.39) |
| 2021 | 0.37 (0.35;0.40) |

Marginal estimates for the association between age and mental healthcare costs by year. As plotted in figure 2.

# A8 Regression output for mental healthcare costs and age – excluding 2021

|  | Coefficient | Robust std. err. | t | P>\|t\| | [95% conf. interval] |  |
| --- | --- | --- | --- | --- | --- | --- |
| Urbanicity |  |  |  |  |  |  |
| 2 | 0,214617 | 0,03224 | 6,66 | 0 | 0,151332 | 0,277903 |
| 3 | 0,40628 | 0,039729 | 10,23 | 0 | 0,328293 | 0,484267 |
| 4 | 0,668468 | 0,035837 | 18,65 | 0 | 0,598121 | 0,738814 |
| 5 | 0,883537 | 0,037469 | 23,58 | 0 | 0,809986 | 0,957088 |
| Sex | 0,190619 | 0,00772 | 24,69 | 0 | 0,175465 | 0,205773 |
| Age |  |  |  |  |  |  |
| 18-34 years | 0,216549 | 0,015442 | 14,02 | 0 | 0,186236 | 0,246861 |
| Year |  |  |  |  |  |  |
| 2016 | 0,008335 | 0,004746 | 1,76 | 0,079 | -0,00098 | 0,017651 |
| 2017 | 0,045222 | 0,004828 | 9,37 | 0 | 0,035745 | 0,054699 |
| 2018 | 0,093269 | 0,005183 | 17,99 | 0 | 0,083095 | 0,103444 |
| 2019 | 0,128209 | 0,006442 | 19,9 | 0 | 0,115563 | 0,140855 |
| 2020 | 0,155967 | 0,006513 | 23,95 | 0 | 0,143182 | 0,168751 |
| age3#year |  |  |  |  |  |  |
| 18-34 years#2016 | 0,005051 | 0,007466 | 0,68 | 0,499 | -0,0096 | 0,019705 |
| 18-34 years#2017 | 0,031696 | 0,008062 | 3,93 | 0 | 0,01587 | 0,047521 |
| 18-34 years#2018 | 0,06769 | 0,008107 | 8,35 | 0 | 0,051777 | 0,083603 |
| 18-34 years#2019 | 0,113135 | 0,010336 | 10,95 | 0 | 0,092846 | 0,133425 |
| 18-34 years#2020 | 0,143896 | 0,008416 | 17,1 | 0 | 0,127376 | 0,160417 |
| constant | -0,61601 | 0,030848 | -19,97 | 0 | -0,67657 | -0,55546 |

Regression output the linear regression of mental healthcare costs and age excluding the year 2021 weighted for the number of insured years per unit of analysis and accounting for urbanicity, sex, year, the interactions between age and year, and for clustering by 3-number postal

# A9 Marginsplot for predicted mental healthcare costs and age – excluding 2021


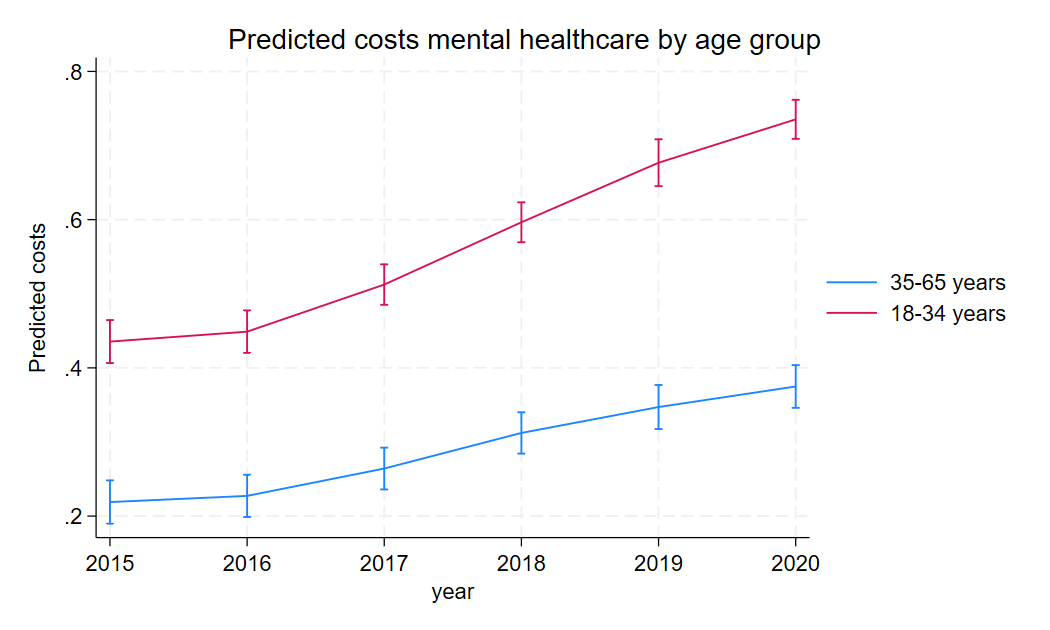


Predicted mental healthcare costs by age group accounting for urbanicity, sex, year, the interaction between age and year. The y-axis shows the log-transformed standardised costs. The X-axis shows the calendar year.

# A10 Marginal estimates for mental healthcare costs by age group - excluding 2021

|  | Age 18-35 | Age 35-65 |
| --- | --- | --- |
|  | *Marginal estimate (95%-CI)* | |
| 2015 | 0.44 (0.41;0.46) | 0.22 (0.19;0.25) |
| 2016 | 0.45 (0.42;0.48) | 0.23 (0.21;0.26) |
| 2017 | 0.51 (0.49;0.54) | 0.26 (0.24;0.29) |
| 2018 | 0.60 (0.57;0.62) | 0.31 (0.28;0.34) |
| 2019 | 0.68 (0.65;0.71) | 0.35 (0.32;0.38) |
| 2020 | 0.74 (0.71;0.76) | 0.37 (0.35;0.40) |

Marginal estimates for mental healthcare costs by age group per year. As plotted in A9.

# A11 Regression output for medical specialist costs and age

|  | Coefficient | Robust std. err. | t | P>\|t\| | [95% conf. interval] |  |
| --- | --- | --- | --- | --- | --- | --- |
| Urbanicity |  |  |  |  |  |  |
| 2 | 0,07005 | 0,015434 | 4,54 | 0 | 0,039753 | 0,100346 |
| 3 | 0,10675 | 0,016951 | 6,3 | 0 | 0,073475 | 0,140025 |
| 4 | 0,14591 | 0,016484 | 8,85 | 0 | 0,113552 | 0,178267 |
| 5 | 0,09393 | 0,024269 | 3,87 | 0 | 0,046291 | 0,141568 |
| Sex | 0,519476 | 0,004642 | 111,9 | 0 | 0,510363 | 0,528588 |
| Age |  |  |  |  |  |  |
| 18-34 years | -0,91014 | 0,009394 | -96,88 | 0 | -0,92858 | -0,8917 |
| Year |  |  |  |  |  |  |
| 2016 | 0,112931 | 0,003137 | 36 | 0 | 0,106772 | 0,119089 |
| 2017 | 0,116198 | 0,003514 | 33,07 | 0 | 0,1093 | 0,123096 |
| 2018 | 0,149116 | 0,003778 | 39,47 | 0 | 0,1417 | 0,156532 |
| 2019 | 0,17737 | 0,004061 | 43,68 | 0 | 0,169398 | 0,185341 |
| 2020 | 0,073577 | 0,004619 | 15,93 | 0 | 0,064511 | 0,082643 |
| 2021 | 0,216505 | 0,004652 | 46,54 | 0 | 0,207373 | 0,225636 |
| age#year |  |  |  |  |  |  |
| 18-34 years#2016 | -0,03846 | 0,005153 | -7,46 | 0 | -0,04858 | -0,02834 |
| 18-34 years#2017 | -0,03288 | 0,005484 | -6 | 0 | -0,04365 | -0,02212 |
| 18-34 years#2018 | -0,03613 | 0,005519 | -6,55 | 0 | -0,04696 | -0,0253 |
| 18-34 years#2019 | -0,05306 | 0,006018 | -8,82 | 0 | -0,06488 | -0,04125 |
| 18-34 years#2020 | -0,0473 | 0,006697 | -7,06 | 0 | -0,06044 | -0,03415 |
| 18-34 years#2021 | -0,06021 | 0,006876 | -8,76 | 0 | -0,07371 | -0,04672 |
| constant | -0,55615 | 0,014705 | -37,82 | 0 | -0,58502 | -0,52729 |

Regression output for the linear regression of medical specialist costs and age weighted for the number of insured years per unit of analysis and accounting for urbanicity, sex, year, the interactions between age and year, and for clustering by 3-number postal code

# A12 Marginsplot for medical specialist costs and age


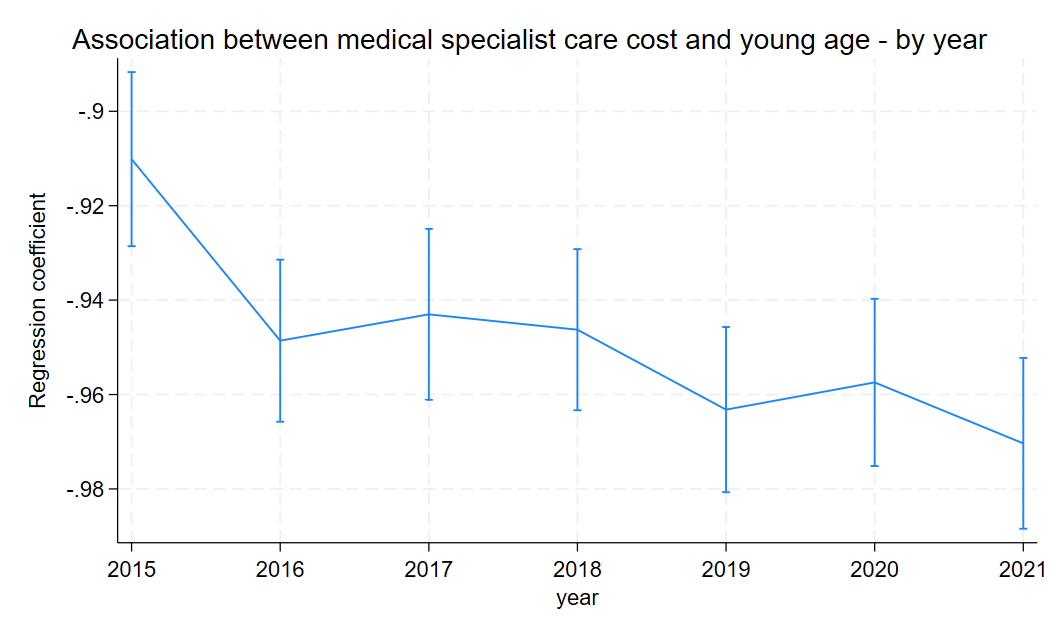


Association between medical specialist care and young age by year. The Y-axis shows the regression coefficient for age. The X-axis shows the calendar year.

# A13 Marginal estimates for the association between medical specialist costs and young age

|  | *Marginal estimate (95%-CI)* |
| --- | --- |
| 2015 | -0.91 (-0.93;-0.89) |
| 2016 | -0.95 (-0.97; -0.93) |
| 2017 | -0.94 (-0.96; -0.92) |
| 2018 | -0.95 (-0.96; -0.93) |
| 2019 | -0.96 (-0.98; -0.95) |
| 2020 | -0.96 (-0.98; -0.94) |
| 2021 | -0.97 (-0.99; -0.95) |

Marginal estimates for the association between medical specialist costs and young age by year. As plotted in A12

# A14 Regression output for general practitioner costs and age

|  | Coefficient | Robust std. err. | t | P>\|t\| | [95% conf. interval] |  |
| --- | --- | --- | --- | --- | --- | --- |
| Urbanicity |  |  |  |  |  |  |
| 2 | -0,00347 | 0,035928 | -0,1 | 0,923 | -0,07399 | 0,067056 |
| 3 | 0,013589 | 0,041538 | 0,33 | 0,744 | -0,06795 | 0,095126 |
| 4 | 0,099502 | 0,039401 | 2,53 | 0,012 | 0,022159 | 0,176845 |
| 5 | 0,095305 | 0,048938 | 1,95 | 0,052 | -0,00076 | 0,191368 |
| Sex | 0,632673 | 0,006582 | 96,12 | 0 | 0,619753 | 0,645592 |
| Age |  |  |  |  |  |  |
| 18-34 years | -0,73446 | 0,013807 | -53,2 | 0 | -0,76156 | -0,70736 |
| Year |  |  |  |  |  |  |
| 2016 | 0,146217 | 0,004677 | 31,27 | 0 | 0,137037 | 0,155396 |
| 2017 | 0,250325 | 0,005518 | 45,37 | 0 | 0,239494 | 0,261156 |
| 2018 | 0,452228 | 0,007656 | 59,07 | 0 | 0,4372 | 0,467255 |
| 2019 | 0,673195 | 0,008566 | 78,59 | 0 | 0,656381 | 0,690009 |
| 2020 | 0,868065 | 0,00943 | 92,05 | 0 | 0,849555 | 0,886576 |
| 2021 | 1,048468 | 0,010321 | 101,59 | 0 | 1,028209 | 1,068727 |
| age#year |  |  |  |  |  |  |
| 18-34 years#2016 | -0,05504 | 0,005106 | -10,78 | 0 | -0,06507 | -0,04502 |
| 18-34 years#2017 | -0,05843 | 0,006214 | -9,4 | 0 | -0,07063 | -0,04624 |
| 18-34 years#2018 | -0,01865 | 0,007075 | -2,64 | 0,009 | -0,03253 | -0,00476 |
| 18-34 years#2019 | 0,02211 | 0,007069 | 3,13 | 0,002 | 0,008234 | 0,035987 |
| 18-34 years#2020 | 0,069346 | 0,008865 | 7,82 | 0 | 0,051944 | 0,086748 |
| 18-34 years#2021 | 0,108135 | 0,009785 | 11,05 | 0 | 0,088927 | 0,127342 |
| Constant | -0,76698 | 0,032271 | -23,77 | 0 | -0,83032 | -0,70363 |

Regression output for the linear regression of general practitioner costs and age weighted for the number of insured years per unit of analysis and accounting for urbanicity, sex, year, the interactions between age and year, and for clustering by 3-number postal code

# A15 Marginsplot for general practitioner costs and age


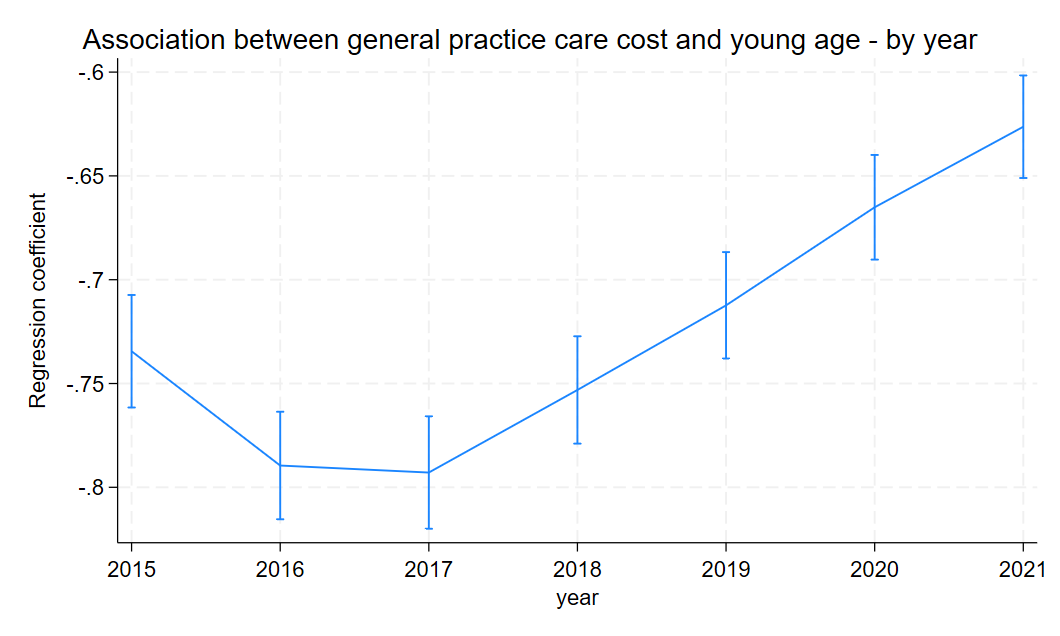


Association between general practitioner costs and young age by year. The Y-axis shows the regression coefficient for age. The X-axis shows the calendar year.

# A16 Marginal estimates for the association between general practitioner costs and young age

|  | *Marginal estimate (95%-CI)* |
| --- | --- |
| 2015 | -0.73 (-0.76;-0.71) |
| 2016 | -0.79 (-0.82; -0.76) |
| 2017 | -0.79 (-0.82; -0.77) |
| 2018 | -0.75 (-0.78 ; -0.73) |
| 2019 | -0.71 (-0.74 ; -0.69) |
| 2020 | -0.67 (-0.69 ; -0.64) |
| 2021 | -0.63 (-0.65; -0.60) |

Marginal estimates for the association between general practitioner costs and young age by year. As plotted in A15.

# A17 Regression output for mental healthcare costs and sex

|  | Coefficient | Robust std. err. | t | P>\|t\| | [95% conf. interval] |  |
| --- | --- | --- | --- | --- | --- | --- |
| stedc |  |  |  |  |  |  |
| 2 | 0,205418 | 0,031246 | 6,57 | 0 | 0,144085 | 0,266752 |
| 3 | 0,393199 | 0,038732 | 10,15 | 0 | 0,317169 | 0,469229 |
| 4 | 0,654857 | 0,035132 | 18,64 | 0 | 0,585895 | 0,723818 |
| 5 | 0,870537 | 0,03663 | 23,77 | 0 | 0,798633 | 0,94244 |
| age3 | 0,291968 | 0,012972 | 22,51 | 0 | 0,266504 | 0,317431 |
| sex |  |  |  |  |  |  |
| V | 0,133419 | 0,0089 | 14,99 | 0 | 0,115949 | 0,150888 |
| year |  |  |  |  |  |  |
| 2016 | 0,004185 | 0,00533 | 0,79 | 0,433 | -0,00628 | 0,014648 |
| 2017 | 0,029849 | 0,005789 | 5,16 | 0 | 0,018484 | 0,041213 |
| 2018 | 0,075407 | 0,005945 | 12,69 | 0 | 0,063738 | 0,087075 |
| 2019 | 0,116338 | 0,007944 | 14,65 | 0 | 0,100745 | 0,131931 |
| 2020 | 0,152889 | 0,0071 | 21,53 | 0 | 0,138953 | 0,166826 |
| 2021 | -0,15761 | 0,007675 | -20,54 | 0 | -0,17267 | -0,14254 |
| sex#year |  |  |  |  |  |  |
| V#2016 | 0,011174 | 0,005632 | 1,98 | 0,048 | 0,00012 | 0,022229 |
| V#2017 | 0,050997 | 0,006528 | 7,81 | 0 | 0,038182 | 0,063812 |
| V#2018 | 0,07979 | 0,006879 | 11,6 | 0 | 0,066287 | 0,093292 |
| V#2019 | 0,09813 | 0,006834 | 14,36 | 0 | 0,084716 | 0,111545 |
| V#2020 | 0,102096 | 0,006882 | 14,84 | 0 | 0,088587 | 0,115605 |
| V#2021 | 0,114878 | 0,007072 | 16,24 | 0 | 0,100996 | 0,12876 |
| _cons | -0,40968 | 0,026957 | -15,2 | 0 | -0,46259 | -0,35676 |

Regression output for the linear regression of mental healthcare costs and sex weighted for the number of insured years per unit of analysis and accounting for urbanicity, age, year, the interaction between sex and year, and for clustering by 3-number postal code

# A18 Marginal estimates for mental healthcare costs by sex

|  | Male | Female |
| --- | --- | --- |
|  | *Marginal estimate (95%-CI)* | |
| 2015 | 0.23 (0.20;0.26) | 0.36 (0.34;0.38) |
| 2016 | 0.23 (0.20;0.26) | 0.38 (0.35;0.40) |
| 2017 | 0.26 (0.23;0.29) | 0.44 (0.42;0.47) |
| 2018 | 0.30 (0.27;0.33) | 0.52 (0.49;0.54) |
| 2019 | 0.34 (0.31;0.38) | 0.58 (0.55;0.60) |
| 2020 | 0.38 (0.35;0.41) | 0.62 (0.59;0.64) |
| 2021 | 0.07 (0.04;0.10) | 0.32 (0.30;0.34) |

Marginal estimates for mental healthcare costs by sex per year. As plotted in figure 3.

# A19 Regression output for mental healthcare costs and the interaction between age and sex

|  | Coefficient | Robust std. err. | t | P>\|t\| | [95% conf. interval] |  |
| --- | --- | --- | --- | --- | --- | --- |
| Urbanicity |  |  |  |  |  |  |
| 2 | 0,205465 | 0,031236 | 6,58 | 0 | 0,144149 | 0,26678 |
| 3 | 0,393278 | 0,038729 | 10,15 | 0 | 0,317255 | 0,4693 |
| 4 | 0,654852 | 0,035117 | 18,65 | 0 | 0,585918 | 0,723785 |
| 5 | 0,86976 | 0,036567 | 23,79 | 0 | 0,797982 | 0,941539 |
| Age |  |  |  |  |  |  |
| 18-34 years | 0,155553 | 0,016357 | 9,51 | 0 | 0,123445 | 0,18766 |
| Year |  |  |  |  |  |  |
| 2016 | 0,002085 | 0,006125 | 0,34 | 0,734 | -0,00994 | 0,014107 |
| 2017 | 0,026893 | 0,006473 | 4,15 | 0 | 0,014187 | 0,0396 |
| 2018 | 0,06567 | 0,006879 | 9,55 | 0 | 0,052168 | 0,079172 |
| 2019 | 0,093349 | 0,007697 | 12,13 | 0 | 0,07824 | 0,108459 |
| 2020 | 0,123208 | 0,007983 | 15,43 | 0 | 0,107538 | 0,138879 |
| 2021 | -0,1855 | 0,008311 | -22,32 | 0 | -0,20182 | -0,16919 |
| age#year |  |  |  |  |  |  |
| 18-34 years#2016 | 0,007918 | 0,010676 | 0,74 | 0,459 | -0,01304 | 0,028874 |
| 18-34 years#2017 | 0,011419 | 0,01083 | 1,05 | 0,292 | -0,00984 | 0,032677 |
| 18-34 years#2018 | 0,033038 | 0,011029 | 3 | 0,003 | 0,011389 | 0,054688 |
| 18-34 years#2019 | 0,072772 | 0,012457 | 5,84 | 0 | 0,04832 | 0,097224 |
| 18-34 years#2020 | 0,092744 | 0,011735 | 7,9 | 0 | 0,069709 | 0,115779 |
| 18-34 years#2021 | 0,087832 | 0,012427 | 7,07 | 0 | 0,063438 | 0,112226 |
| Sex |  |  |  |  |  |  |
| Female | 0,093144 | 0,009288 | 10,03 | 0 | 0,074913 | 0,111376 |
| age#sex |  |  |  |  |  |  |
| 18-34 years#V | 0,122636 | 0,012114 | 10,12 | 0 | 0,098858 | 0,146415 |
| year#sex |  |  |  |  |  |  |
| 2016#V | 0,01261 | 0,007069 | 1,78 | 0,075 | -0,00127 | 0,026486 |
| 2017#V | 0,036731 | 0,007491 | 4,9 | 0 | 0,022028 | 0,051435 |
| 2018#V | 0,055251 | 0,00803 | 6,88 | 0 | 0,039487 | 0,071014 |
| 2019#V | 0,069339 | 0,007759 | 8,94 | 0 | 0,054109 | 0,08457 |
| 2020#V | 0,065577 | 0,008251 | 7,95 | 0 | 0,04938 | 0,081774 |
| 2021#V | 0,065578 | 0,008243 | 7,96 | 0 | 0,049398 | 0,081758 |
| age#year#sex |  |  |  |  |  |  |
| 18-34 years#2016#V | -0,00572 | 0,012446 | -0,46 | 0,646 | -0,03015 | 0,018712 |
| 18-34 years#2017#V | 0,041296 | 0,012589 | 3,28 | 0,001 | 0,016584 | 0,066008 |
| 18-34 years#2018#V | 0,07064 | 0,012848 | 5,5 | 0 | 0,045421 | 0,095859 |
| 18-34 years#2019#V | 0,082368 | 0,013132 | 6,27 | 0 | 0,056589 | 0,108146 |
| 18-34 years#2020#V | 0,104271 | 0,01418 | 7,35 | 0 | 0,076437 | 0,132105 |
| 18-34 years#2021#V | 0,140948 | 0,014653 | 9,62 | 0 | 0,112184 | 0,169712 |
| Constant | -0,36478 | 0,027118 | -13,45 | 0 | -0,41801 | -0,31155 |

Regression output for the linear regression of mental healthcare costs and age weighted for the number of insured years per unit of analysis and accounting for urbanicity, sex, year, the interactions between age,year and, and for clustering by 3-number postal code

# A20 Marginal estimates for the association between mental healthcare costs and young age by gender

|  | Male | Female |
| --- | --- | --- |
|  | *Marginal estimate (95%-CI)* | |
| 2015 | 0.15 (0.12;0.19) | 0.28 (0.25;0.31) |
| 2016 | 0.16 (0.13;0.19) | 0.28 (0.25;0.31) |
| 2017 | 0.17 (0.14;0.20) | 0.33 (0.30;0.36) |
| 2018 | 0.19 (0.16;0.22) | 0.38 (0.35;0.41) |
| 2019 | 0.23 (0.20;0.26) | 0.43 (0.40;0.46) |
| 2020 | 0.25 (0.22;0.28) | 0.48 (0.45;0.50) |
| 2021 | 0.24 (0.22;0.27) | 0.51 (0.48;0.53) |

Marginal estimates for the association between young age and mental healthcare costs by year and sex. As plotted in figure 4.
